# Gut-Brain Psychology: Rethinking Psychology From the Microbiota–Gut–Brain Axis

**DOI:** 10.3389/fnint.2018.00033

**Published:** 2018-09-11

**Authors:** Shan Liang, Xiaoli Wu, Feng Jin

**Affiliations:** ^1^Key Laboratory of Mental Health, Institute of Psychology, Chinese Academy of Sciences, Beijing, China; ^2^University of Chinese Academy of Sciences, Beijing, China

**Keywords:** gut-brain psychology, microbiota–gut–brain axis, diet, modernization, lifestyle, superorganism, mental disorders, nutritional psychology

## Abstract

Mental disorders and neurological diseases are becoming a rapidly increasing medical burden. Although extensive studies have been conducted, the progress in developing effective therapies for these diseases has still been slow. The current dilemma reminds us that the human being is a superorganism. Only when we take the human self and its partner microbiota into consideration at the same time, can we better understand these diseases. Over the last few centuries, the partner microbiota has experienced tremendous change, much more than human genes, because of the modern transformations in diet, lifestyle, medical care, and so on, parallel to the modern epidemiological transition. Existing research indicates that gut microbiota plays an important role in this transition. According to gut-brain psychology, the gut microbiota is a crucial part of the gut-brain network, and it communicates with the brain via the microbiota–gut–brain axis. The gut microbiota almost develops synchronously with the gut-brain, brain, and mind. The gut microbiota influences various normal mental processes and mental phenomena, and is involved in the pathophysiology of numerous mental and neurological diseases. Targeting the microbiota in therapy for these diseases is a promising approach that is supported by three theories: the gut microbiota hypothesis, the “old friend” hypothesis, and the leaky gut theory. The effects of gut microbiota on the brain and behavior are fulfilled by the microbiota–gut–brain axis, which is mainly composed of the nervous pathway, endocrine pathway, and immune pathway. Undoubtedly, gut-brain psychology will bring great enhancement to psychology, neuroscience, and psychiatry. Various microbiota-improving methods including fecal microbiota transplantation, probiotics, prebiotics, a healthy diet, and healthy lifestyle have shown the capability to promote the function of the gut-brain, microbiota–gut–brain axis, and brain. It will be possible to harness the gut microbiota to improve brain and mental health and prevent and treat related diseases in the future.

## Current Challenges in Psychology

**FIGURE 1:**
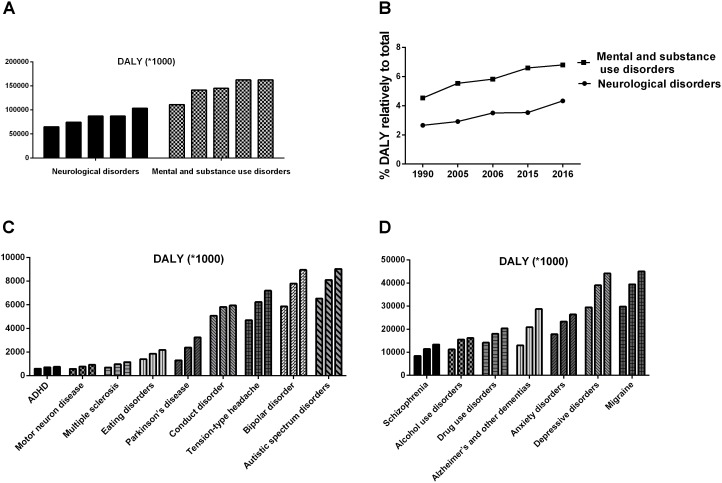
The ever-increasing medical burden induced by mental disorders and neurological diseases (DALYs and Collaborators, 2016, 2017). **(A,B)** Shows the disability-adjusted life years (DALYs) induced by mental disorders and neurological diseases, respectively. **(C,D)** Shows the DALYs induced by different diseases in 1990, 2006, and 2016.

Psychology is a discipline that targets the rules of human psychological phenomena and behavior. Unfortunately, it seems like the more we know about human psychology, the more we realize we do not know. Until recently, not a single mental disorder had an established definite biomarker, either physiological, biochemical, or genetic. The application of psychology seems to lag behind other disciplines, and mental illnesses remain medical challenges. In the last few decades, the number of patients with mental disorders and neurologic diseases has increased rapidly, causing a great escalation of medical burden, as shown in **Figure 1** (DALYs and Collaborators, 2016, 2017; GBD 2017 Neurological Disorders Collaborator Group, 2017). Although the overall medical burden created by mental disorders exceeded one-fifth of the total, the rates of treatment and recovery were far below those of other diseases (Ledford, 2014; Smith and Torres, 2014). All of these findings suggest that the existing research has neglected the fact that the human being is a superorganism.

## Re-Recognizing the Subject of Psychology: the Superorganism

Following the development of bioinformatics and gut microbiota research in the 21st century, scientists have found that the human being is a superorganism carrying billons of microorganisms, such as bacteria, archaea, fungi, viruses, and protozoa, living on its internal and external surfaces (Group, 2008; Limon et al., 2017; Manrique et al., 2017; Wampach et al., 2017). These microorganisms mostly inhabit the skin surface and digestive, respiratory, urinary, and reproductive tracts. Microorganisms amounting to more than 1 kg inhabit the digestive tract; these are considered to be the most important microorganisms in the human body, and they are collectively called the gut microbiota. The microbiota contains 300–3,000 different species, whose total number exceeds 10^14^, which is almost 10 times the total number of human cells; the genes encoded therein surpass 5 million, which is more than 200 times the number of human genes (Human Microbiome Project Consortium, 2012; Burcelin et al., 2013; Sandoval-Motta et al., 2017). Since 2008, *Nature* has advocated calling the human being “we” rather than “I” because more than 90% of the total cells and genes of the superorganism are microorganisms. In daily life, individuals living together for a long time usually share many similarities. This phenomenon could be mainly due to the convergence of people’s microorganisms (Yatsunenko et al., 2012), since most of human genes are relatively stable after birth. These microorganisms have established interdependent and mutualistic relationships with humans over the long process of evolution; therefore, they are called commensal microbiota (Fraune and Bosch, 2010).

### Maturation and Function of the Superorganism Are Modulated by the Commensal Microbiota

The growth and development of human beings are not only modulated by their own genes, but they are also influenced by their commensal microbiota. Humans provide living space and food for the microbiota and unconsciously regulate the composition and number of microorganisms, while the microbiota impacts the maturation and function of human beings (Ulvestad, 2009). Embryonic development is influenced by maternal gut microbiota, and the development and function of most mammalian systems are also affected by their own commensal microbiota after birth (Manco, 2012; McFall-Ngai et al., 2013).

The gut microbiota is involved in the metabolism and digestive absorption of nutrients; it aids in the digestion of resistant carbohydrates, the decomposition of endogenous and exogenous proteins, the degradation of bile acid, and the synthesis of vitamins and other bioactive compounds (Nicholson et al., 2012; Cabral, 2013). The colonization of the gut microbiota is indispensable for the maturation of the immune system, and its impact is likely to have a critical period, which means that the colonization only works in the critical period and cannot be remedied after that time (Gensollen et al., 2016; Knoop et al., 2017). The maturation of the neuroendocrine system is also regulated by the gut microbiota, and this influence may have a critical period, too (Sudo et al., 2005; Sudo, 2014). Both the maturation and function of the brain and mind are impacted by the gut microbiota (Diaz Heijtz et al., 2011; Borre et al., 2014; Desbonnet et al., 2014; Parashar and Udayabanu, 2016; Vuong et al., 2017).

Meanwhile, the respiratory tract microbiota plays an important role in the development and function of the respiratory system, and abnormal microbiota may be involved in the occurrence of respiratory disease (Man et al., 2017). The skin microbiota not only constitutes the first biological barrier of the organism, but also influences immune function, and skin microbiota abnormalities are closely related to skin disorders, including eczema and psoriasis (Egert et al., 2017). Finally, healthy vaginal microbiota is necessary for female health, and it is beneficial for the development of commensal microbiota in the offspring (Humphries, 2017).

In conclusion, individual maturation and function are strongly linked to commensal microbiota (Collins et al., 2012; McFall-Ngai et al., 2013). Acquiring adequate microbiota can help ensure a healthy and happy human life. When the microbiota is deprived, dysfunction can appear in the digestive system, immune system, endocrine system, nervous system, and even in behavior and cognition (Sudo et al., 2005; Fang and Evans, 2013; Crumeyrolle-Arias et al., 2014).

### Dramatic Changes in the Superorganism in Modern Society

Human society has changed significantly since the industrial revolution, which was followed by tremendous variations in diet, lifestyle, and health care. Although the genes of the human have not changed much, the important component of the superorganism microbiota has undergone tremendous change (Gomez, 2017; Mancabelli et al., 2017). Remote rural areas have experienced relatively small variation over the past century, with the inhabitants having gut microbiota distinct from those of modern city dwellers. Even in developed countries, rural–urban differences in gut microbiota exist. For example, the bacteria that metabolize fiber have decreased, whereas the bacteria that metabolize animal protein and fat have increased in city dwellers; even in people who have moved from villages to cities, the gut microbiota seems to have changed to a more urbanized microbiota (De Filippo et al., 2017). Modernization has been changing the microbiota by various means, including diet, lifestyle, and medication (De Filippo et al., 2017; Gomez, 2017; Mancabelli et al., 2017).

#### Diet Changes

Diet shapes the gut microbiota, and different foods prompt the proliferation of different microorganisms (Duncan et al., 2007; Wu et al., 2011; Voreades et al., 2014; Shanahan et al., 2017; Singh et al., 2017). Even short-term dietary changes alter the human microbiota (David et al., 2014; Li J. et al., 2017). The human diet—including dietary structure, dietary habits, and food processing—has experienced great changes following modernization, and these alterations have significantly influenced the gut microbiota (Zarrinpar et al., 2014; Roca-Saavedra et al., 2017; Statovci et al., 2017).

In terms of dietary structure, refined carbohydrates dominate the total food intake; the intake of meat, fat, sugar, and salt has increased rapidly, whereas the intake of dietary fibers has decreased sharply. However, high-fat diets and high refined carbohydrate diets, which are rich in sucrose and fructose, perturb the gut microbiota (Hu et al., 2014; Magnusson et al., 2015; Rosas-Villegas et al., 2017). Dietary fibers, which include β-glucan, arabinoxylans, and resistant starch, are non-digestible polysaccharides that are abundant in whole grains, functionally known as microbiota-accessible carbohydrates (MACs) (Daien et al., 2017; Gong et al., 2018). They are the main source of energy for gut bacteria, and they are essential to maintain human health (Koh et al., 2016; Daien et al., 2017; Williamson, 2017). A long-term low-MAC diet has been found to lead to microbiota extinction, which presented intergenerational effects. The gut microbiota was restored by a high-fiber diet in the first generation, but it was not restored in the subsequent generations (Sonnenburg et al., 2016).

In terms of dietary habits, the number of times people eat at home has reduced significantly, whereas the number of times people eat out and eat snacks has increased rapidly. In food processing, the proportion of fresh food and traditional fermented food has decreased significantly, whereas the proportion of processed food and industrially produced food has increased rapidly (De Filippo et al., 2017; Derrien and Veiga, 2017; Statovci et al., 2017). The food additives, pesticide residues, and drug residues in the food could greatly disrupt the gut microbiota upon entry into the digestive tract (Suez et al., 2014; Chassaing et al., 2015, 2017; Bian et al., 2017; Jiang et al., 2017; Roca-Saavedra et al., 2017). Although most standard additives are harmless to the body, they have effects on the gut microbiota, which have generally been ignored until recently (Roca-Saavedra et al., 2017). In addition to antiseptics such as potassium sorbate and sodium benzoate, other additives also significantly perturb the gut microbiota. Emulsifiers, including hydroxymethyl cellulose and polysorbate 80, damage the gut microbiota and induce inflammation and metabolic syndrome (Chassaing et al., 2015). Artificial sweeteners, such as saccharin, aspartame, and sucralose, alter the gut microbiota and gut-brain function, inducing glucose intolerance (Suez et al., 2014; Bian et al., 2017).

In short, it has become increasingly difficult for a person to acquire adequate commensal microbiota from food, and the significant changes in diet in recent decades may be the cause of the convergent evolution of the gut microbiota in the modern urban population (De Filippo et al., 2017; Derrien and Veiga, 2017).

#### Lifestyle Changes

Human beings live in a bacterial world, and lifestyle factors such as environment and habit determine the species and the number of bacteria that one carries (Rook, 2009; Shanahan, 2009; De Filippo et al., 2017; Jin et al., 2017; Velmurugan et al., 2017). The living environment has changed since modernization. More and more people have migrated from villages to cities and/or shifted from working outdoors to working indoors; as a result, there are fewer and fewer opportunities for people to touch pollution-free soil and water to acquire harmless microorganisms (Rook, 2009; Shanahan, 2009; Jin et al., 2017; Velmurugan et al., 2017). Lifestyle habits have changed as well. Instead of delivering babies vaginally, modern pregnant women more frequently undergo cesarean sections. Additionally, modern mothers often do not have enough time to breastfeed for a variety of reasons, such as work, so their children are usually fed processed formula milk powder. Moreover, physical activity levels have been significantly reduced with the convenience of modern life. The circadian rhythm is also often disrupted; the average sleep duration has decreased, and day and night inversion has become increasingly common. The changes in delivery mode, feeding patterns, physical activity, and circadian rhythm could all impact the commensal microbiota (Shanahan, 2009; Di Mauro et al., 2013; Hallgren et al., 2016; Khalyfa et al., 2017; Paschos and FitzGerald, 2017; Zhao and Zhang, 2017).

#### Health Care Changes

Health care conditions have been greatly improved since modernization, but overtreatment and excessive hygiene have perturbed the commensal microbiota (Armelagos, 2009; Rook, 2009; Blaser, 2016; Two et al., 2016; Joshipura et al., 2017; Rook et al., 2017). Although drugs, including antibiotics, may be harmless to the human body, they can damage the commensal microbiota (Blaser, 2016). As public health standards have been enhanced, disinfection and sterilization have become more and more common in the workplace and at home. Personal hygiene standards have also risen; the frequency of brushing teeth, washing hands, and washing clothes have increased, which means greater daily use of chemical products and more and more overly clean people (Two et al., 2016; Joshipura et al., 2017).

### Great Transformation of Disease Types

In brief, the tremendous changes in diet, lifestyle, and health care have deprived modern people of opportunities to gain adequate environmental and foodborne microorganisms. All of these factors have changed the superorganism. The biggest alterations in the human body from the times of the agricultural society to the industrial society may not be in the human genes, but in the commensal microbiota with which we coexist (Gomez, 2017; Mancabelli et al., 2017). The symbiotic relationship between humans and microorganisms has been established over millions of years of evolution by natural selection, and it is relatively exclusive. For example, only the gut microbiota of mice can facilitate their own immune maturation, whereas those of humans and rats cannot (Ferreira and Veldhoen, 2012). The new human gut microbiota was not established through long-term natural selection, and it easily conflicts with the human body. Thus, more and more human diseases have appeared that deviate from Hardy-Weinberg Equilibrium, and they cannot be explained by the genes present in humans alone (Lerner et al., 2017; Neish et al., 2017; Rook et al., 2017).

The changes in the species and the construction of the commensal microbiota inevitably cause alterations in human function. For example, more and more modern city dwellers present intolerance to traditional foods, including gluten, milk, and eggs (Derrien and Veiga, 2017; Skypala, 2017; Tordesillas et al., 2017). While traditional infectious diseases that prevailed in the agricultural society have rapidly decreased, autoimmune diseases, such as allergies and asthma; cardiovascular diseases, such as hypertension; metabolic diseases, including diabetes and fatty liver; mental disorders, including depression and anxiety; and neurodegenerative diseases, such as Alzheimer’s disease and Parkinson’s disease, have all increased significantly. This is the epidemiological transition that modern people are experiencing (Armelagos et al., 2005; Becker, 2007; Rook and Lowry, 2008; Armelagos, 2009; Elliott and Weinstock, 2009; Bloomfield, 2013; DALYs and Collaborators, 2017).

The most complex and important component of the commensal microbiota is the gut microbiota, which is one of the most biodiverse ecosystems in the world (Montiel-Castro et al., 2013). The existence and construction of this ecosystem are closely related to human health and disease. It is believed that the microbiota plays a crucial role in the pathophysiology of digestive diseases, metabolic diseases, immune diseases, and neurodevelopmental diseases (Backhed, 2010; Clemente et al., 2012; Eloe-Fadrosh and Rasko, 2013). Targeted therapy of the gut microbiota will be an important and promising field in the future (Petrof et al., 2013; Wallace and Redinbo, 2013; Young, 2017).

## Gut Microbiota, Gut-Brain, and Gut-Brain Psychology

### Gut-Brain

The gut is the biggest digestive organ, immune organ, and endocrine organ of the human body, and it also possesses a nervous system [the enteric nervous system (ENS)], which is relatively independent of the brain. During the fetal period, neural crest cells almost simultaneously differentiate into the central nervous system (CNS) and ENS. The ENS presents many similarities with the brain in terms of neuronal components, neurotransmitters, and functional independence (Petrof et al., 2013; Wallace and Redinbo, 2013; Young, 2017). The gut is a microbial organ with 90–95% of its total cell number consisting of microorganisms. The gut provides living space and food for microorganisms, while the microbiota influences the development and function of the gut. The gut and gut microbiota work together to perform the tasks of digestion, immune and endocrine functions, and neurotransmission (Forsythe et al., 2010; Lyte, 2010; Nicholson et al., 2012; Sudo, 2014; Ridaura and Belkaid, 2015). We call this microbial organ gut-brain because, unlike other peripheral organs, it can work without instructions from the brain, and this specificity can easily be found in the persistent vegetative state (Liang et al., 2012; Liang et al., 2018a,b). The gut-brain not only completes its local function, but also regulates human behavior and cognition, similar to the brain (Grenham et al., 2011; Collins et al., 2012; Mayer et al., 2014a; Foster et al., 2016). Gut-brain psychology is the discipline of studying the relationship between the gut-brain and mind. Research in this field has increased rapidly over the last decade.

### Gut Microbiota Regulates the Development of Brain and Behavior

**FIGURE 2:**
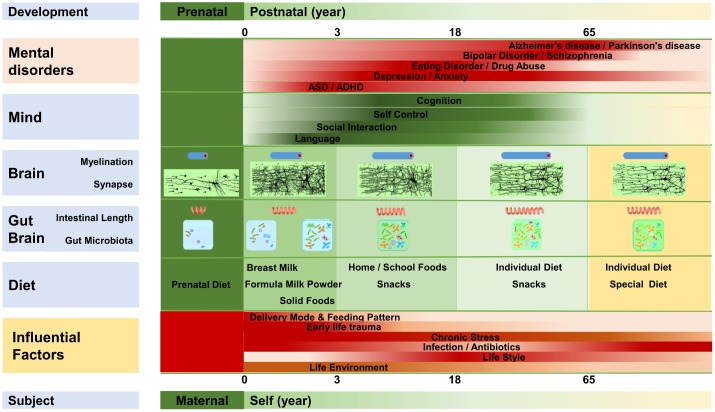
The gut-brain, brain, and mentality develop almost synchronously throughout the lifespan. The gut-brain, brain, and mentality undergo similar developmental patterns; all three are susceptible to several factors that influence the gut microbiota. Myelination, intestinal length, and the gut microbiota develop almost synchronously. Diet plays an important role in the maturation of the gut-brain and brain, and mentality is regulated by the development of the brain and gut-brain. Microbiota disruption at different stages is likely to increase the incidence of different mental disorders.

As shown in **Figure 2**, the gut microbiota develops almost simultaneously with the brain and psychology. It not only regulates the structure and function of the gut-brain, but also influences the development of the brain and behavior (Luczynski et al., 2016; Sharon et al., 2016; Kundu et al., 2017; Vuong et al., 2017; Carlson et al., 2018), and microbiota disturbances at different stages can induce different brain and mental disorders (Borre et al., 2014; Gur et al., 2015; Sampson and Mazmanian, 2015; Dinan and Cryan, 2017).

The human gut microbiota does not appear suddenly, but experiences a gradual growth from simple to complex, then tends to stabilize, and finally declines slowly (Garcia-Pena et al., 2017; Vuong et al., 2017). The fetus probably starts to come in contact with the microorganisms early in the womb, at which time the microbiota is mainly determined by maternal physiological and psychological conditions, diet, drugs, and so forth (Lim et al., 2016). The early microbiota of the newborn is largely determined by the delivery mode. Vigorous newborns typically gain many microorganisms, such as *Lactobacillus*, from the maternal vagina, whereas neonates born by cesarean gain microorganisms, such as *Clostridium*, from the air and the maternal skin (Penders et al., 2006; Bokulich et al., 2016; von Mutius, 2017). Feeding patterns regulate the microbiota in the next stage. Breastfed infants obtain more *Bifidobacterium* and *Lactobacillus*, whereas infants fed with formula milk obtain more *Enterococcus* and *Enterobacterium* (Penders et al., 2006; Bokulich et al., 2016; Kundu et al., 2017). Antibiotic use also reduces the abundance of *Bifidobacterium* and *Bacteroides*, and it delays the development of gut microbiota (Penders et al., 2006; Bokulich et al., 2016; Wampach et al., 2017). Afterward, the gut microbiota develops with age and dietary changes. For example, the original dominant species such as *Bifidobacterium* decrease with age (Penders et al., 2006; Bokulich et al., 2016). The phylogenetic composition of the infant microbiota increases rapidly after birth, and it evolves toward an adultlike configuration within a 3-year period (Yatsunenko et al., 2012; Bokulich et al., 2016). Then, the phylogenetic composition and diversity continue to evolve, and the drastic changes of adolescence greatly impact the development of the microbiota (Kundu et al., 2017). The gut microbiota is relatively stable in adulthood, and more than 60% of the microbiota, including Bacteroidetes and Actinobacteria, experiences little changes (Faith et al., 2013; Borre et al., 2014). In old age, the diversity of the gut microbiota declines, while the richness of some opportunistic pathogens, including some *Clostridium* species, increases (Claesson et al., 2011; Kundu et al., 2017).

The gut microbiota influences the development and maturation of the brain and mind (Diaz Heijtz et al., 2011; Borre et al., 2014; Galland, 2014; Mu et al., 2016; Manderino et al., 2017; Bruce-Keller et al., 2018). Germ-free (GF) animals not only present developmental defects in brain structure, but also show abnormal mental development (Diaz Heijtz et al., 2011; Desbonnet et al., 2014; Ogbonnaya et al., 2015; Hoban et al., 2016; Luczynski et al., 2016; Chen et al., 2017). Both neuroplasticity and myelin plasticity are influenced by the gut microbiota (Ogbonnaya et al., 2015; Hoban et al., 2016). An abnormal gut microbiota can induce brain dysfunction and mental disorders. Risk factors disturbing microbiota growth, such as maternal stress, early infection, antibiotic use, and early adversity, also perturb the development of the brain and mind (O’Mahony et al., 2009; Borre et al., 2014; Gur et al., 2015; Diaz Heijtz, 2016; Lim et al., 2016; Slykerman et al., 2017). Since early postnatal life is the critical stage for the development of the gut-brain, brain, and mind, microbiota abnormality at this time could lead to irreversible damage in the brain and mind (Borre et al., 2014; Bokulich et al., 2016). This could be a part of the reason why early adversity increases susceptibility to mental disorders (O’Mahony et al., 2009, 2017; Mika et al., 2017).

### Normal Psychology and Behavior Cannot Exist Without Gut Microbiota

The gut microbiota plays a significant role in the host’s mind and behavior, although this role is usually ignored (Luczynski et al., 2016; Vuong et al., 2017).

First, the gut microbiota regulates pain perception and influences visceral pain response and peripheral pain response. The visceral pain sensitivity of GF mice was shown to be increased and alleviated after transplantation with the feces microbiota of wild-type mice (Luczynski et al., 2017). Pain sensitivity is also augmented after antibiotic treatment or infection, and reduced after supplementation with certain probiotics (Vuong et al., 2017). Myalgic encephalomyelitis/chronic fatigue syndrome patients present abnormal gut microbiota and metabolomics (Armstrong et al., 2017), and their symptoms can be alleviated after treatment with certain probiotics (Rao et al., 2009). Many pain-related disorders, such as functional abdominal pain, migraine, and chronic back pain, are strongly linked with abnormal microbiota (Gawronska et al., 2007; Albert et al., 2013; Dai et al., 2017).

Second, cognitive functions, including learning capacity and memory, are closely related to the gut microbiota (Gareau, 2016; Manderino et al., 2017). Either depriving the commensal microbiota or disrupting the microbiota with antibiotics damages working memory and spatial memory, whereas probiotic administration improves memory (Liang et al., 2015; Ohsawa et al., 2015; Wang et al., 2015; Vuong et al., 2017).

Third, mood and emotion are affected by the gut microbiota (Luczynski et al., 2016; Cowan et al., 2017; Hoban et al., 2017; Vuong et al., 2017). Germ-free animals present abnormal anxiety-like behaviors, which appear to be amenable to microbial intervention (Luczynski et al., 2016). Pathogen infection quickly induces sickness behavior, with infected subjects showing fatigue, social avoidance, decreased appetite, and increased anxiety-like behavior (Lyte et al., 2006; Lyte, 2013; De Palma et al., 2014; Gur et al., 2015). Perturbing the gut microbiota using stress or antibiotics also increases anxiety-like and depression-like behaviors (Lurie et al., 2015; Frohlich et al., 2016; Slykerman et al., 2017). Meanwhile, supplementing with certain probiotics, prebiotics, or fermented foods reduces negative behaviors and improves these emotions (Cryan and Dinan, 2012; Steenbergen et al., 2015; McKean et al., 2017; Vuong et al., 2017).

Fourth, temperament and character are closely linked with the gut microbiota—they can even transmit from one subject to another through fecal microbiota transplantation (FMT) under certain conditions (Collins et al., 2013; Kelly et al., 2016; Zheng et al., 2016; Kim H.N. et al., 2017). The transfer of microbiota from high anxiety-like Balb/C mouse strain to low anxiety-like GF NIH Swiss mice has been found to be anxiogenic in the recipient. The same is true of the reverse transplantation: NIH Swiss bacteria transferred into GF Balb/C mice attenuate the recipients’ anxious phenotype (Bercik et al., 2011a; Collins et al., 2013). Among toddlers, surgency/extraversion is positively associated with phylogenetic diversity (Christian et al., 2015). Among adults, high neuroticism and low conscientiousness are correlated with the high abundances of Gammaproteobacteria and Proteobacteria, respectively. Meanwhile, high conscientiousness is associated with an increased abundance of some universal butyrate-producing bacteria, including *Lachnospiraceae* (Kim H.N. et al., 2017).

Fifth, stress management is impacted by the gut microbiota. The gut microbiota is a part of the stress response system (Dinan and Cryan, 2012; Luczynski et al., 2016). Psychological stresses not only activate the neuroendocrine, immune, and nervous systems, but they also destroy mood and disturb the gut microbiota (Gur et al., 2015; Liang et al., 2015; Bharwani et al., 2016). The amygdala, which plays a crucial part in stress-related mood and behavior response as well as in emotion regulation, is remarkably impacted by the gut microbiota (Cowan et al., 2017; Hoban et al., 2017). A healthy microbiota helps the host to cope with stress, whereas an abnormal microbiota reduces the resistance and increases the susceptibility to stress-related disorders (Moloney et al., 2014; Parashar and Udayabanu, 2016; Cowan et al., 2017; Vuong et al., 2017).

Sixth, gut microbiota affects dietary behavior. The dietary patterns of mammals are strongly linked to their gut microbiota, which vary significantly among animals with different dietary compositions (Nishida and Ochman, 2017). A typical example of this is the red panda, in whose gut the metabolism of dietary bamboo depends on the microbiota (Kong et al., 2014). The human appetite is probably modulated by the gut microbiota; some food tastes good possibly because the microbiota requires suitable food to promote its proliferation (van de Wouw et al., 2017). The gut microbiota may also play a vital part in eating disorders, such as anorexia nervosa (Glenny et al., 2017; van de Wouw et al., 2017).

Finally, social interaction and reproductive behavior are strongly linked with the commensal microbiota. A normal gut microbiota is essential for the development of social behavior (Desbonnet et al., 2014). Germ-free mice present more social avoidance, while microbiota reconstitution in time improves their social interaction (Montiel-Castro et al., 2013; Desbonnet et al., 2014; Buffington et al., 2016). Social anxiety in response to novel subjects or a novel environment is also related to the gut microbiota (Parashar and Udayabanu, 2016). Mating choices that are dependent on olfaction and reproductive behavior in mammals are impacted by the commensal microbiota (McFall-Ngai et al., 2013; Stumpf et al., 2013).

### Both Mental Illnesses and Neurological Diseases Are Closely Related to Abnormal Microbiota

Research indicates that mental disorders are likely to be rooted in abnormal gut microbiota, and targeting the microbiota should play a vital role in future therapy (Fond et al., 2015; Liang et al., 2015, 2018b; Ochoa-Reparaz and Kasper, 2018). Depressive disorder is strongly linked to the gut microbiota (Jiang et al., 2015; Liang et al., 2018b), and depressive symptoms can be transmitted from humans to GF or microbiota-depleted rodents through FMT (Kelly et al., 2016; Zheng et al., 2016), while probiotics intervention can alleviate and improve the disorder (Liang et al., 2015; Pirbaglou et al., 2016; Wallace and Milev, 2017). The gut microbiota also plays a crucial part in the etiology of anxiety disorders, such as obsessive compulsive disorder, post-traumatic stress disorder, and panic attacks, while regulating the microbiota brings about therapeutic effects for these disorders (Kantak et al., 2014; Leclercq et al., 2016; Schnorr and Bachner, 2016; Turna et al., 2016). Bipolar disorder is significantly related to microbiota abnormalities (Evans S.J. et al., 2017; Yolken and Dickerson, 2017), and microbiota regulation probably alleviates the disorder (Hamdani et al., 2015; Dickerson et al., 2017). Schizophrenia is related with the dysfunction of microbiota–gut–brain axis (Nemani et al., 2015; Rodrigues-Amorim et al., 2018; Shen et al., 2018), and improving the gut-brain and immune functions by targeting the microbiota could possibly produce beneficial effects (Davey et al., 2013; Tomasik et al., 2015; Dickerson et al., 2017). Additionally, patients subjected to neurodevelopmental disorders, including autism spectrum disorders (ASD) and attention deficit hyperactivity disorder (ADHD), possess abnormal gut microbiota (Mayer et al., 2014b; Aarts et al., 2017; Strati et al., 2017), while correcting the microbiota abnormalities in a timely manner probably improves the development of the brain and behavior and has remedial effects (Borre et al., 2014; Partty et al., 2015; Kang et al., 2017; Doenyas, 2018; Yang et al., 2018). Neurodegenerative diseases, such as Alzheimer’s disease and Parkinson’s disease, may also originate from the gut (Hu et al., 2016; Liddle, 2018); aberrations first appear in the gut microbiota and gut (Li W. et al., 2017; Mancuso and Santangelo, 2017), with ENS degeneration occurring first and gradually spreading to the CNS, while improving gut-brain function by adjusting the microbiota has remedial effects (Hu et al., 2016; Liddle, 2018). The gut microbiota is also involved in the pathophysiology of behavior disorders, including drug addiction and substance abuse, while behavior modifications combined with microbiota regulation may have beneficial effects (Engen et al., 2015; Vogtmann et al., 2015; Kiraly et al., 2016; Skosnik and Cortes-Briones, 2016). Additionally, the gut microbiota plays a vital role in the pathophysiology of neurobiological diseases, such as multiple sclerosis, hepatic encephalopathy, epilepsy, and migraine (Liang et al., 2012; Sharon et al., 2016; Wu et al., 2016; Dai et al., 2017; van den Hoogen et al., 2017). The prevalence of mental disorders and neurological diseases has been ever increasing, almost in parallel with the changes in the human commensal microbiota. In response to this, therapies targeting the microbiota have gained more and more attention, and attempts to treat these diseases by microbiota intervention using probiotics, prebiotics, and FMT have increased steadily (Cryan and Dinan, 2012; Dinan et al., 2013; Liang et al., 2015; Pirbaglou et al., 2016; He et al., 2017; Kang et al., 2017; Mika et al., 2017; Zhao et al., 2017; Bruce-Keller et al., 2018; Yang et al., 2018). Thus far, researchers have proposed several theories, such as the gut microbiota hypothesis (Liang et al., 2018a,b), the “old friends” hypothesis (Rook and Lowry, 2008), and the leaky gut theory (Smythies and Smythies, 2014), to explain the relationship between the gut microbiota and the above-mentioned diseases.

#### The Gut Microbiota Hypothesis of Brain Disorders

In 2002, F Jin’s lab found that pigs fed with *Lactobacillus*-fermented fodder were more resistant to swine influenza and porcine reproductive and respiratory syndrome when compared with conventional pigs. Their meat was more nutritious and delicious and even their characters were more meek and less aggressive. Since then, the lab has turned its attention to the relationship of the commensal microbiota with behavior and psychology. In 2012, the lab tried to comprehensively elucidate the role that commensal microbiota plays in human mental disorders and neurological diseases (Liang et al., 2012). Then, after a series of experiments, the lab found that anxiety-like behavior, depression-like behavior, and cognitive impairment induced by gastrointestinal disease, a high-fat diet, and antibiotic use were all associated with gut microbiota abnormalities and improved by gut microbiota regulation using specific *Lactobacillus* strains (Hu et al., 2014, 2015; Luo et al., 2014; Wang et al., 2015). Their next study indicated that the key reason for both acquired and inborn depression was likely to be an abnormal gut microbiota (some of the data were unpublished). In the chronic restraint stress depression model, depressive rats had microbiota that was different from control rats; the traditional antidepressant citalopram alleviated some behavioral and physiological aberrations, but could not recover the microbiota, while the *Lactobacillus helveticus* NS8 intervention not only improved the behavioral and physiological abnormalities, but also recovered the microbiota (Liang et al., 2015). In the inborn depression model, the Wistar Kyoto (WKY) rats possessed a gut microbiota that was distinct from the control Wistar rats. Chronic restraint stress aggravated the depressive-like symptoms, and *Lactobacillus helveticus* NS8 supplementation presented the opposite effect with stress; it also alleviated the behavioral, biochemical, and microbiota aberrations as in the case of the acquired depression model. The lab further found that aggressive behavior was connected with the microbiota; for example, prisoners with violent tendencies presented higher levels of blood ammonia (NH_3_) (Duan et al., 2015). They also found that ASD, ADHD, and Tourette syndrome were all closely related with gut microbiota abnormalities and could be improved by specific probiotic intervention. This research is still ongoing. Patients diagnosed with Alzheimer’s disease and Parkinson’s disease have also been found to possess abnormal gut microbiota, and their hypofunction in the brain and gut-brain can be improved by specific probiotic intervention (Hu et al., 2016; Li W. et al., 2017). Based on the above research and observations, the lab proposed the gut microbiota hypothesis (Liang et al., 2018a,b). According to this hypothesis, many factors in the modern society, including unhealthy diet, antibiotic use, and life stress, disturb the gut microbiota, and an abnormal microbiota may be a direct risk factor for mental and brain illnesses. Abnormal microbiota and the subsequent dysfunction in the microbiota–gut–brain axis are the main pathophysiology of these disorders, and regulating the microbiota by valid methods, such as probiotics or a healthy diet, will have therapeutic effects.

#### The “Old Friends” Hypothesis

The “old friends” hypothesis, or the early immune challenge hypothesis, was proposed by Rook on the basis of Strachan’s hygiene hypothesis (Strachan, 1989; Rook and Lowry, 2008; Kramer et al., 2013; Rook, 2013). This theory proposes that the symbiotic relationship between humans and the commensal microbiota has been formed over millions of years of evolution, and that it is evolution dependent and adapted to the hunter-gatherer life. This microbiota was or used to be humans’ “old friends,” which include microorganisms and helminths found in pollution-free water, soil, and food. However, in modern society, dramatic changes in health care, lifestyle, and diet have greatly diminished exposure to these friends, which has resulted in abnormalities in immune development. Only through adequate exposure to these “old friends” can naïve dendritic cells (DCs) mature to regulatory dendritic cells (DCreg). In turn, the DCreg induce the maturation of T-lymphocytes into regulatory T-lymphocytes (Treg). The Treg regulate immune tolerance information, which means that these “old friends” and human tissues do not generate an immune response. Regulatory T-lymphocytes also regulate the intensity of immune response by certain biological process, such as the release of interleukin 10 (IL-10), and avoid excessive immune responses that could damage the human body. However, with the lack of the “old friends,” the DCs cannot mature, and T-lymphocytes differentiate into effective T-lymphocytes, such as Th1, Th2, and Th17. In this condition, subjects may present immune responses to harmless microorganisms and their own tissues, as in the case of allergies and autoimmune diseases, and they are also likely to present inappropriate and uncontrollable inflammation. Chronic inflammation may be a risk factor for many diseases, including allergies, autoimmune diseases, chronic inflammatory diseases, and mental disorders (Becker, 2007; Rook and Lowry, 2008; Elliott and Weinstock, 2009; Rook et al., 2011, 2017; Bloomfield, 2013; Kramer et al., 2013).

#### The Leaky Gut Theory

The human body has two major barriers—the gut barrier and the blood–brain barrier (BBB)—in addition to the placental barrier in pregnant females. The gut barrier regulates the flow of nutrients and signal molecules in the body and prevents the entry of microorganisms, food residue, and harmful substances. The BBB controls the entry and exit of substances in the circulatory system, and its key components are tight junctions (TJs). Thus, the integrity of the barriers is critical for human health (Borre et al., 2014; Kelly et al., 2015). The gut microbiota regulates the development and function of these barriers, for example, influencing the formation of TJs (Braniste et al., 2014). Many factors, such as stress, alcohol use, unhealthy diet, and heavy metal, damage the gut barrier, increase intestinal permeability, and allow biomacromolecules and microorganisms to pass through to the body that could not do so before; this syndrome is called leaky gut (Leclercq et al., 2012; Slyepchenko et al., 2017). The early leaky gut theory emphasizes more on the nutrient absorption and immune function of gut barrier (Fink, 1990), whereas the latest leaky gut theory posits that when the gut barrier is broken, not only bacterial translocation, circulating lipopolysaccharides (LPS) levels, and immunoglobulin (Ig) M and IgA levels increase, but the BBB is also impaired, and cyclic biomacromolecules can even pass through the BBB, reaching the brain and inducing neuroinflammation (McCusker and Kelley, 2013; Smythies and Smythies, 2014; Kelly et al., 2015). These are key contributors to many inflammatory diseases, metabolic diseases, mental disorders, and neurological diseases (de Kort et al., 2011; Fasano, 2012; Smythies and Smythies, 2014; Potgieter et al., 2015; Garcia Bueno et al., 2016; Slyepchenko et al., 2017). Repairing the gut barrier by microbiota regulation is likely to be an effective therapy for these diseases (Ait-Belgnaoui et al., 2012).

In conclusion, human mind and behavior are not only regulated by the brain, but are also probably impacted by the gut-brain. Thus, factors perturbing the gut microbiota also affect the brain and mind simultaneously. Although each of the above theories has a different focus, all of them hold that mental disorders are closely related to abnormal gut microbiota. The “old friends” hypothesis emphasizes the evolution of the microbiota, whereas the leaky gut theory emphasizes on the function of the gut barrier, but both the theories recognize immune dysfunction as the main cause and future therapy target for brain disorders. Both the gut microbiota hypothesis and the leaky gut theory hold that abnormal microbiota injures gut-brain function, thereby, damaging brain function and finally inducing mental and brain disorders. However, the gut microbiota hypothesis puts more emphasis on how the microbiota influences the brain and behavior, which is currently the field of greatest concern in gut-brain psychology.

## Gut Microbiota Impacts the Brain and Behavior Through the Microbiota–Gut–Brain Axis

**FIGURE 3:**
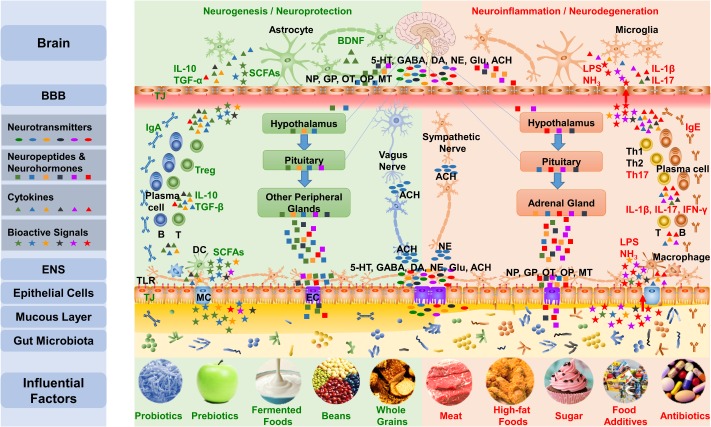
The gut-brain communicates with the brain mainly via three pathways of the microbiota–gut–brain axis. First, the gut microbiota influences the synthesis and secretion of neurotransmitters, including 5-HT, GABA, DA, NE, Glu, and ACh. Gut signals can activate the ENS and primary afferents as well as transmit messages to the brain through the vagus nerve and the sympathetic nervous system. Second, the gut microbiota impacts the concentration and function of neuropeptides that include neuropeptides, gut peptides, OT, and opioid peptides and neurohormones, such as melatonin, communicating with the brain through the neuroendocrine pathway, including the HPA axis and hypothalamus-pituitary-other peripheral glands axis. Third, the gut microbiota regulates the function of TJs and TLRs in the gut barrier and BBB, adjusts the differentiation of lymphocytes, and impacts the brain via the immune pathway. Additionally, the bioactive products of the microbiota also influence the microbiota–gut–brain axis. The five influential factors on the left protect the microbiota and the mucous layer, leading to the production of beneficial substances, such as SCFA, by the microbiota, which results in an anti-inflammatory environment; meanwhile, the five factors on the right are likely to interrupt the normal function of the gut-brain, which can induce mucus loss and microbiota disturbance, leading to the production of harmful substances such as lipopolysaccharide and NH_3_ and resulting in a pro-inflammatory environment. NP, Neuropeptides; GP, Gut peptides; OP, Opioid peptides; MT, Melatonin; LPS, lipopolysaccharide; MC, M cell; EC, Enteroendocrine cell.

The brain-gut axis is the bidirectional communication between the CNS and the digestive tract (Wood, 2007; O’Mahony et al., 2011; Liang et al., 2012). Recent research reminds us to bring gut microbiota into this axis, which is the microbiota–gut–brain axis. As shown in **Figure 3**, the influences of microbiota overstep the gut and reach the whole body, especially the brain, through the microbiota–gut–brain axis (Cryan and O’Mahony, 2011; Grenham et al., 2011; Collins et al., 2012; Mayer et al., 2014a, 2015; Smith, 2015; Kelly et al., 2017; Cox and Weiner, 2018; Dinan et al., 2018; Liang et al., 2018b). The microbiota–gut–brain axis mainly contains three pathways: the nerve pathway, neuroendocrine pathway, and immune pathway.

### Nerve Pathway

The nerve pathway is the fastest way through which microbiota affects the brain and behavior; it includes neural conduction (Forsythe et al., 2014), neurotransmitter (Wall et al., 2014), neurogenesis (Ogbonnaya et al., 2015), apoptosis, and neurodegeneration (Thion et al., 2017; Westfall et al., 2017).

#### Neural Conduction

The oral microbiota impacts not only lower organs, but also the head and brain (Montiel-Castro et al., 2013; Gholizadeh et al., 2016). In certain conditions, the oral microbiota and its metabolites can even directly activate cranial nerves, such as the olfactory nerve and trigeminal nerve, to influence the brain and behavior; they are probably involved in the pathophysiology of neurodegenerative diseases (Garcia-Pena et al., 2017; Marizzoni et al., 2017).

The gut microbiota can affect the brain and behavior through the vagus nerve (Forsythe and Kunze, 2012; Quigley, 2017; Bonaz et al., 2018). The sickness behavior induced by infection is closely related to the vagus nerve. The primary afferents send out the microbial signals first and activate the vagus nerve quickly and then transmit the message to the brain (Forsythe et al., 2014). The gut microbiota recognizes neural signals released by the host and responds appropriately to prompt its own proliferation. They can also respond quite differently to different catecholamines and recognize other neural signals, such as serotonin (5-HT), gamma-aminobutyric acid (GABA), and neuropeptides (Lyte, 2014b; Sudo, 2014; Wall et al., 2014). Infection by pathogens, such as *Campylobacter jejuni* and *Citrobacter amalonaticus*, increases anxiety-like behavior, while probiotic supplementation with bacteria, including *Lactobacillus rhamnosus* and *Bifidobacterium longum*, reduces anxiety-like and depression-like behaviors. Both the anxiogenic/depressive effect and the anxiolytic/antidepressant effects depend on the vagus nerve, and they are eliminated by vagotomy (Lyte et al., 2006; Bercik et al., 2011b; Bravo et al., 2011; Forsythe and Kunze, 2012; Forsythe et al., 2014).

It is unclear how the sympathetic nervous system works in the communication between the gut and brain, but the research on hypertension has revealed that it is involved in the regulation of the gut barrier function (Taylor and Takemiya, 2017; Yang and Zubcevic, 2017).

#### Neurotransmitters

The neurotransmitter content of mammals is regulated not only by their own bodies, but also by their gut microbiota (Diaz Heijtz et al., 2011; Wall et al., 2014). Gut microbiota can synthesize neurotransmitters directly (Lyte, 2014a; Wall et al., 2014; Mazzoli and Pessione, 2016), and more than 90% of 5-HT and 50% of dopamine (DA) are synthesized in the gut (Sudo, 2014; Yano et al., 2015). Many bacteria, including *Bacillus* strains and some lactic acid bacteria (LAB) species, synthesize catecholamines and/or acetylcholine (ACh) (Wall et al., 2014). Members of the genera *Candida*, *Streptococcus*, *Escherichia*, and *Enterococcus* synthesize 5-HT (Holzer and Farzi, 2014). Several coryneform bacteria and many LAB strains are able to produce glutamate (Glu), and both prokaryotes and eukaryotes synthesize GABA through the decarboxylation of Glu by glutamate decarboxylase (Mazzoli and Pessione, 2016).

Gut microbiota regulates the synthesis of neurotransmitters by changing the neurotransmitter-related metabolism pathways (O’Mahony et al., 2014; Mazzoli and Pessione, 2016). Taking 5-HT as an example, more than 90% of the body’s 5-HT is synthesized in the gut by enterochromaffin cells (Margolis et al., 2014). Tryptophan can synthesize 5-HT, and it can also produce kynurenine catalyzed by the largely hepatic-based enzyme tryptophan-2,3-dioxygenase (TDO) or the ubiquitous indoleamine-2,3-dioxygenase (IDO) (O’Mahony et al., 2014). Some indigenous spore-forming microbes can induce the biosynthesis and bioavailability of 5-HT by prompting enterochromaffin cells to upregulate tryptophan hydroxylase 1 expression (Yano et al., 2015; Yang et al., 2017). The gut microbiota also regulates the brain concentration of 5-HT, which is probably implemented by the inhibition of the kynurenine pathway (Desbonnet et al., 2008; Luo et al., 2014; Liang et al., 2015; Kennedy et al., 2017).

The gut microbiota impacts the expression of neurotransmitter-related genes. Many genes linked with neurotransmitters in the striatum of male GF NMRI mice were found to be expressed differently when compared with their specific pathogen-free (SPF) counterparts (Diaz Heijtz et al., 2011). Additionally, the mRNA expression of the NR2B subunit of *N*-methyl-D-aspartate (NMDA) receptors in the central amygdala and the 5-HT_1A_ receptor in the hippocampus of female NMRI mice was reduced when compared with the SPF mice (Neufeld et al., 2011). Moreover, the gene expression of GABA_A_ and GABA_B_ in several areas of the brain changed after *Lactobacillus rhamnosus* JB-1 intervention in adult BALB/c mice (Bravo et al., 2011).

#### Neurogenesis, Apoptosis, and Neurodegeneration

Adult neurogenesis is influenced by the gut microbiota. The hippocampal neurogenesis of adult GF mice was found to be increased when compared with conventional mice, and postweaning microbial colonization of GF mice did not affect the abnormalities, suggesting that there is a critical window in early life during which microbial colonization influences adult hippocampal neurogenesis (Ogbonnaya et al., 2015). Brain neurogenesis is regulated by the brain-derived neurotrophic factor (BDNF), while the content and gene expression of BDNF are regulated by the gut microbiota. The concentration and gene expression of brain BDNF changed in GF animals and decreased in frail subjects, but increased after probiotic intervention (Sudo et al., 2005; Bercik et al., 2010; Ait-Belgnaoui et al., 2014; Distrutti et al., 2014; Maqsood and Stone, 2016).

Myelination and myelin plasticity are also impacted by the gut microbiota. The absence of microbiota was found to result in hypermyelinated axons in the prefrontal cortex and the overexpression of myelin component genes in a region- and sex-dependent manner. Conventional microbiota colonization following weaning inhibited the overexpression of these genes, but it did not reverse the levels of the proteins they encoded (Hoban et al., 2016). The gut microbiota plays a vital role in diseases characterized by CNS demyelination, such as multiple sclerosis (Lee et al., 2011; Girolamo et al., 2017; van den Hoogen et al., 2017).

The apoptosis of neurons is related to gut microbiota. The programmed cell death in the hypothalamus and some hippocampus areas was found to be increased in neonatal GF mice when compared with conventional mice, which was paralleled by the elevation in the number and density of microglia and the increase of microglia biomarkers in these areas (Castillo-Ruiz et al., 2018).

Neurodegeneration and its accumulation are influenced by the gut microbiota. Abnormal microbiota or microbial metabolites may influence neurodegeneration through the promotion of amyloid formation by human proteins or by enhancing inflammatory responses to endogenous neuronal amyloids (Friedland and Chapman, 2017; Hoffman et al., 2017; Minter et al., 2017), while microbiota interventions, such as probiotic supplementation, may prevent or even reverse this process (Hu et al., 2016; Westfall et al., 2017).

### Endocrine or Humoral Pathway

The gut is the largest endocrine organ in the human body. It contains more than 20 different kinds of enteroendocrine cells, expresses more than 30 kinds of hormone genes, each with several phenotypes, and secretes more than 100 hormonal peptides (Raybould, 2010; Rehfeld, 2014). The gut microbiota can influence the brain and behavior through the endocrine pathway, directed by the neuroendocrine cells, neuropeptides, and neuroactive substances (Liang et al., 2012; Holzer and Farzi, 2014; Sudo, 2014; Wall et al., 2014).

#### Neuroendocrine

The endocrine system also governs the whole body via signaling molecules. The hypothalamus is a higher nervous center for the regulation of viscera and endocrine function; it plays a vital role in the secretion of pituitary hormones and autonomic nervous system activity. The pituitary gland is the most important endocrine gland and the main regulator of homeostasis through hormones. It controls the synthesis of many key hormones that regulate metabolism, development, reproduction, and other functions. The hypothalamus, pituitary gland, and peripheral endocrine glands constitute the neuroendocrine system, which facilitates the communication between the brain and gut (Sudo, 2014; Rieder et al., 2017; Cussotto et al., 2018).

The hypothalamic–pituitary–adrenal (HPA) axis is the main pathway of neuroendocrine transmission, and it is a crucial part of the stress response system. The gut microbiota is essential to the development and function of the HPA axis (Liang et al., 2012; Sudo, 2014). The absence of a microbiota induces abnormal development of the HPA axis and only colonization within a critical window will prompt HPA axis maturation (Sudo et al., 2005; Liang et al., 2012; Keightley et al., 2015). Germ-free mice were found to present an enhanced HPA stress response and reduced sensitivity to negative feedback signals when compared with SPF mice (Sudo et al., 2005; Sudo, 2006, 2014). The exaggerated HPA response by GF mice was reversed by reconstitution with *Bifidobacterium infantis*, but it was exacerbated by mono-colonization with enteropathogenic *Escherichia coli*. The enhanced HPA response of GF mice was also partly corrected by reconstitution with SPF feces at an early stage, but it was not corrected by any reconstitution performed at a later stage (Sudo et al., 2005; Sudo, 2014). Even if one has an intact microbiota, many factors, such as chronic stress and antibiotic use, can impair the function of the HPA axis, while probiotic intervention improves the function of the HPA axis both in juveniles and adults (Eutamene and Bueno, 2007; Gareau et al., 2007; Liang et al., 2015; Wang et al., 2015).

The gut microbiota also plays an important role in the activity of other peripheral endocrine glands, such as the release of sex hormones and thyroid hormones (Neuman et al., 2015; Cussotto et al., 2018). The gut microbiota regulates estrogen primarily through the secretion of β-glucuronidase, which can deconjugate estrogen into its active forms (Baker et al., 2017). Testosterone enriches the male microbiota with specific bacteria, including SFB, *E. coli*, and *Shigella*-like bacteria, while the transplantation of the male microbiota into females elevates testosterone levels (Markle et al., 2013).

#### Neuropeptides and Neurohormones

Neuropeptides are a type of bioactive peptide that are widespread in the nervous system; they include neuropeptide Y (NPY), oxytocin (OT), calcitonin gene-related peptide, vasoactive intestinal peptide, somatostatin, corticotrophin-releasing factor (CRF) and so forth. These neuropeptides serve as messengers of the microbiota–gut–brain axis and are either released by enterocytes to regulate microbiota or secreted by the microbiota to influence the host (Holzer and Farzi, 2014; Lach et al., 2017).

The gut microbiota regulates the concentration and activity of gut peptides/gut hormones, including insulin, glucagon, gastrin, cholecystokinin, and leptin (Berthoud, 2008; Lach et al., 2017; Worthington et al., 2018). For example, insulin sensitivity is regulated by the gut microbiota; microbiota disturbance induces insulin resistance, whereas microbiota regulation alleviates insulin resistance (Bekkering et al., 2013; He and Shi, 2017; Kim Y.A. et al., 2017).

The NPY family mainly consists of NPY, peptide YY (PYY), and pancreatic polypeptide (PP). Neuropeptide Y is the most abundant neuropeptide in brain, and it is found at all levels of the microbiota–gut–brain axis. Meanwhile, PYY and PP are exclusively expressed by the endocrine cells of the digestive system, but they can pass through the BBB to impact the brain (Holzer et al., 2012). The NPY family plays important roles in regulating energy homeostasis, mood, and stress resilience (Holzer, 2016; Lach et al., 2017). Neuropeptide Y regulates microbiota composition mainly by altering gastrointestinal activity and immunity. In turn, the microbiota recognizes NPYs, impacts their synthesis and secretion, and ultimately influences the brain and behavior (Holzer et al., 2012; Holzer and Farzi, 2014; Holzer, 2016; Lach et al., 2017).

Oxytocin plays an important part in many activities, such as parturition, lactation, social interaction, and stress response (Fineberg and Ross, 2017). The synthesis and release of OT are probably impacted by the gut microbiota. Microbiota depletion from weaning by antibiotics induces abnormal behaviors and cognitive impairment, and reduces OT mRNA levels in the hypothalamus (Desbonnet et al., 2015). Maternal high-fat diet (MHFD) offspring exhibit social deficits, gut microbiota abnormalities, and OT immunoreactive neuron reduction in the hypothalamus; these abnormalities can be prevented by cohousing with the offspring of mothers on a regular diet or *Lactobacillus reuteri* treatment, and can be transmitted to GF mice after microbiota colonization from MHFD mice (Buffington et al., 2016).

Opioid peptides are one kind of neurohormones that widely exist in the brain and peripheral organs (Terenius, 2000; Bodnar, 2016). The endogenous opioid system plays a crucial role in many different kinds of human activities, of which pain and analgesia, tolerance, and dependence are of the greatest concern (Akbarali and Dewey, 2017; Bodnar, 2017; Hearing et al., 2018). Although it is unclear how the endogenous opioid system is involved in the microbiota–gut–brain axis, opioids significantly alter the composition of the gut microbiota, while the microbiota possibly plays important roles in behavioral responses to opioids, including the development of tolerance to its pain-relieving effects (Meng et al., 2015; Banerjee et al., 2016; Kiraly et al., 2016; Lazaro et al., 2016; Skosnik and Cortes-Briones, 2016; Acharya et al., 2017; Akbarali and Dewey, 2017; Wang and Roy, 2017).

Furthermore, circadian rhythms are fundamental properties of mammals, and the microbiota is also regulated by the host’s circadian clock (Paulose and Cassone, 2016; Zhao and Zhang, 2017). Melatonin is a neurohormone secreted by the pineal gland that plays a vital role in the regulation of the circadian rhythm. Host circadian rhythms are influenced by bacterial signaling via the immune system, and the gut bacteria are sensitive to melatonin (Paulose et al., 2016; Thaiss et al., 2017). For example, melatonin specifically increases the magnitude of swarming in cultures of *Enterobacter aerogenes* in a dose- and temperature-dependent manner (Paulose and Cassone, 2016).

#### Neuroactive Substances

The neuroactive substances generated during gut microbiota metabolism, including fatty acids, neural signals, and polyamines, also impact the brain and behavior (Forsythe and Kunze, 2012; Kibe et al., 2014; Wall et al., 2014).

The gut microbiota produces short-chain fatty acids (SCFAs), such as acetic acid, propionic acid, and butyric acid, mostly from the degradation of fibers (Koh et al., 2016). The SCFAs are recognized by the receptors in enterocyte and enteroendocrine cells; thereby, SFCAs further affect the nervous and immune systems (Wall et al., 2014; Koh et al., 2016). In addition, they can pass the BBB to regulate brain development and neurotransmitter synthesis, thereby impacting the maturation and function of microglia (Erny et al., 2015). Polyunsaturated fatty acids (PUFAs), including arachidonic acid and docosatetraenoic acid, are important constituents of the brain, and they affect brain growth and neurotransmission. PUFAs regulate the gut microbiota, while microbiota, such as *Bifidobacterium breve* NCIMB702258, impacts the brain PUFA content (Wall et al., 2012; Robertson et al., 2017). Conjugated linoleic acid (CLA) can pass through the BBB and be metabolized in the brain, thereby influencing the brain and behavior, while certain bacteria, including some strains of *Lactobacillus* and *Bifidobacterium*, synthesize CLA (Wall et al., 2014).

The gut microbiota also produces other substances, such as amines, phenols, NH_3_, indole, indole derivatives, and sulfureted hydrogen, from the metabolism of proteins and amine acids (Hamer et al., 2012; Pimentel et al., 2013; Agus et al., 2018). Take NH_3_ as an example; most of the ammonia in the blood is produced in the gut by the decomposition of urea by gut bacteria, such as *Helicobacter pylori*. The accumulation of NH_3_ in the blood damages astrocyte cells and disturbs neurotransmitters, including Glu and GABA. Hyperammonemia is one of the important risk factors of neurological diseases, such as hepatic encephalopathy and autism (Albrecht, 2007; Liang et al., 2012; Wang et al., 2012).

### Immune Pathway

The gut is also the biggest immune organ of the human body. Its internal surface area is about 200 square meters, and it forms a barrier separating the intestinal tissue from the outside. Gut mucosal immunity is one of the most important parts of the innate immune system, and the immune cells in gut-associated lymphatic tissue (GALT) account for about 70 to 80% of the total immunologically active cells (Tlaskalova-Hogenova et al., 2005). The immune system plays a crucial role in the symbiotic relationship with commensal microbiota; it has coevolved with the microbiota for millions of years, and it cannot mature without normal microbial colonization. The absence of the microbiota leads to significant immune deficiency (Gensollen et al., 2016). The gut microbiota regulates the development and function of innate and adaptive immunity and influences neuroimmunity and inflammation to change the brain and behavior (Freestone et al., 2008; Lee and Mazmanian, 2010; Liang et al., 2012; Lyte, 2014a; Neuman et al., 2015; Levy et al., 2017).

#### Innate Immunity

The maturation and function of the innate immunity depend on the gut microbiota. The gut microbiota affects the development and function and the immune barrier, and it regulates the expression of pattern recognition receptors (PRRs) and the development of innate immune cells (Tlaskalova-Hogenova et al., 2005; Thaiss et al., 2014).

The development and function of the gut barrier and BBB are dependent on the gut microbiota. Barrier deficiency induced by microbiota absence or dysbiosis increases susceptibility to various diseases, including allergies and inflammatory bowel disease (Geuking et al., 2014; Gensollen et al., 2016). The absence of a microbiota downregulates the expression of TJs, increases BBB permeability, and can even induce leaky brain (Braniste et al., 2014; Kelly et al., 2015; Hu et al., 2016). This abnormality could last from the fetal stage to adulthood. Supplementing with specific probiotics has been found to increase the expression of TJs and reduce BBB permeability (Bien-Ly and Watts, 2014; Braniste et al., 2014). Abnormal microbiota not only leads to leaky gut and leaky brain, but it is also likely to induce stress-related disorders and neurodegenerative diseases (Kelly et al., 2015; Hoffman et al., 2017).

The gut microbiota impacts the expression and signal transmission of PRRs (Thaiss et al., 2014). Many PRRs, such as toll-like receptors (TLRs) and nucleotide oligomerization domain-like receptors (NLRs), are expressed in enterocytes; they recognize microbiota material and stimulate a series of immune responses to eliminate or inactivate the pathogen, but tolerate the commensal and harmless material (Artis, 2008; Thaiss et al., 2014). For example, TLR-4 can recognize bacterial LPS and induce immune responses, and the overactivation of TLR-4 in the periphery may play an important part in stress-related mental illnesses and substance abuse (Garcia Bueno et al., 2016). When the gut barrier is impaired, circulating LPS increase and enter into other organs including the brain, resulting in various physiological and/or behavioral symptoms (Rudzki and Szulc, 2018). Lipopolysaccharide injection is a common immune activation model, and systemic LPS treatment might be used as an *in vivo* model for neuroinflammation or neurodegeneration (Noh et al., 2014).

The gut microbiota regulates the differentiation and development of innate immunocytes. Gut innate immunocytes, such as macrophages, innate lymphoid cells, and DC, differentiate and mature following normal microbial colonization (Tlaskalova-Hogenova et al., 2005; Thaiss et al., 2014). Microglia are a kind of highly specialized tissue macrophages in the CNS; they account for 20% of the total neuroglia, and they play a vital role in immune surveillance and homeostasis of the CNS (Prinz et al., 2014). The gut microbiota regulates the maturation and function of neuroglia from prenatal stages in a sex- and time-dependent manner (Erny et al., 2015; Matcovitch-Natan et al., 2016; Thion et al., 2017). The absence of a microbiota, microbiota disturbance, and limited microbiota complexity all induce microglia deficiency, leading to impaired innate immune responses, while recolonization of a normal microbiota partly recovers microglia features in an SCFA-dependent manner (Erny et al., 2015).

#### Adaptive Immunity

Adaptive immunity develops and matures during exposure to and through combat with the microbiota. The gut microbiota regulates the differentiation and function of lymphocytes, influencing the synthesis and release of antibodies (Artis, 2008; Lee and Mazmanian, 2010). The immune system can distinguish beneficial bacteria and pathogens and tolerate self-components and harmless material only if it is exposed to the microbiota in early life (Rook and Lowry, 2008; Knoop et al., 2017).

The differentiation and function of T-lymphocytes are regulated by the gut microbiota (Knoop et al., 2017). The gut microbiota regulates whether naïve CD4+T-lymphocytes (Tnai) differentiate into effective T-lymphocytes to produce a pro-inflammatory response or into Treg to generate anti-inflammatory effects (Honda and Littman, 2016). In conditions that include microbial absence and microbiota abnormality, SFB prompt the Tnai to differentiate into Th17 and induce autoimmune diseases, such as multiple sclerosis (Lee and Mazmanian, 2010; Lee et al., 2011). Even maternal immune activation facilitates the differentiation of Th17, increasing the incidence of neurodevelopmental diseases in the offspring (Kim S. et al., 2017), while probiotic interventions, including *Lactobacillus helveticus* and *Bacteroides fragilis*, induce the differentiation of Treg and the release of IL-10, thereby improving immunity (Ochoa-Reparaz et al., 2009; Ohland et al., 2013; Liang et al., 2015).

The gut microbiota also regulates the differentiation and development of B-lymphocytes, influencing the synthesis and secretion of Ig (Honda and Littman, 2016). Even microbiota metabolites, such as SCFA, influence the differentiation by changing the expression of related genes (Hansson et al., 2011; Kim et al., 2016; McCoy et al., 2017). Immunoglobulin A is a vital component of non-inflammatory immune protection, and it also influences the composition and diversity of the microbiota, while the commensal microbiota regulates the synthesis and release of IgA (Dolle et al., 2016; Honda and Littman, 2016). Commensal microbiota inhibits the secretion of IgE, but promotes the secretion of IgG (McCoy et al., 2017). Absence of a microbiota decreases the content of IgA and IgG1, while increasing the content of IgE, thereby increasing the susceptibility to various diseases (Gensollen et al., 2016).

#### Inflammation

The gut microbiota not only impacts the differentiation and maturation of immune cells, but it also regulates the immune response. A healthy microbiota prompts immunocytes to release moderate anti-inflammatory cytokines, such as IL-10, transforming growth factor beta (TGF-β), and TGF-α, and to secrete moderate pro-inflammatory cytokines, such as IL-1β, IL-17, IFN-γ, and tumor necrosis factor alpha (TNF-α), facilitating the appropriate immune responses (Rook and Lowry, 2008; Rothhammer et al., 2018). However, abnormal microbiota induces an imbalance between anti-inflammation and pro-inflammation, impairs the functions of immune tolerance and immune surveillance, and may even result in chronic inflammation. Chronic inflammation is a key factor in autoimmune diseases and inflammatory diseases, and it can also be found in obese and aging subjects (Rook and Lowry, 2008; Lee and Mazmanian, 2010; Liang et al., 2012).

Abnormal microbiota not only leads to peripheral inflammation, but also probably results in neuroinflammation; it could further damage the BBB integrity, inducing neuron apoptosis, microglia dysfunction, and neurodegeneration, finally causing memory decline, abnormal behavior, and dyskinesia, which are omens of many mental disorders and brain diseases (Daulatzai, 2014; Castanon et al., 2015; Maranduba et al., 2015; Rea et al., 2016; Sampson et al., 2016). Meanwhile, the reconstitution of a healthy microbiota using probiotics or prebiotics is likely to improve immunity and alleviate the brain dysfunction and cognitive and behavioral abnormalities (Dinan et al., 2013; Daulatzai, 2014; Liang et al., 2015; Liu et al., 2015).

### Information Integration in the Microbiota–Gut–Brain Axis

The interplay among the nervous system, endocrine system, and immune system makes the brain and gut-brain multifunctional organs; both the brain and gut-brain orchestrate our metabolism, immunity, and endocrine function. Beyond the comprehensive analysis of the brain and gut-brain, the interaction also appears in information transmission via neuronal, endocrine, and immune pathways. The influence of the gut microbiota on the brain and behavior is the result of this interplay.

The gut-brain integrates the endogenous and exogenous signals of the neuronal, endocrine, and immune pathways, and enterocytes recognize and respond to messages from more than two pathways. For example, the enterochromaffin cell is a crucial endocrine and information conversion cell located on the surface layer of the gut that expresses various receptors, such as 5-HT, norepinephrine, and CRF receptors. When stimulated, it secretes active substances, such as 5-HT and signal peptides, which activate the afferent nerve endings and transmit the message upward through the vagus nerves (Rhee et al., 2009). The synthesis and release of 5-HT by enterochromaffin cells are impacted by the gut microbiota (Yano et al., 2015). Gut epithelial cells and lymphocytes also recognize microbiota signals by PRRs and secrete substances, including cytokines, 5-HT, and CRF, to activate immune, neuronal, and endocrine pathways (Artis, 2008; Rhee et al., 2009).

The three pathways of the microbiota–gut–brain axis interact with each other. For example, the increase of pro-inflammatory cytokines in inflammation enhances the activity of IDO and promotes the metabolization of tryptophan into kynurenine. Meanwhile, under stress condition, the increased glucocorticoid levels heighten TDO activity and induce the kynurenine pathway. Both conditions inhibit 5-HT synthesis by reducing available tryptophan, changing behaviors, and even possibly leading to depression (Le Floc’h et al., 2011; Baganz and Blakely, 2013; O’Mahony et al., 2014).

In conclusion, the microbiota–gut–brain axis is a bidirectional information communication network. The brain governs other organs and regulates the survival and proliferation of the microbiota, while the microbiota impacts the brain and behavior through neuronal, endocrine, and immune pathways. The emotional, behavioral, and brain changes under stress affect the microbiota through the microbiota–gut–brain axis. Hosts can also consciously change their diet to induce the proliferation of beneficial microorganisms in order to improve microbiota–gut–brain axis function and promote health and wellbeing.

## Targeting Microbiota–Gut–Brain Axis to Promote Brain and Mental Health

It is foreseeable that the establishment of gut-brain psychology will bring tremendous changes to psychology and related disciplines. Gut-brain psychology will contribute to the development of general psychology, aiding research on subjects, such as character, memory, and behavior. It may also help illuminate controversial areas, including the study of unconsciousness. However, its more crucial influence is likely to be in clinical application, for example, in regulating the brain and behavior through gut microbiota intervention. The related research and applications will undoubtedly exert a far-reaching impact on many fields, including psychology, medication, food, and environment (Barratt et al., 2017; Leulier et al., 2017; McKenzie et al., 2017).

There are mainly seven recognized microbiota interventions: the GF technique, pathogen infection, antibiotics, FMT, probiotics, prebiotics, and diet (Cryan and Dinan, 2012; Aroniadis and Brandt, 2013; Cammarota et al., 2014; Liu et al., 2015); all of the methods have shown great potential in regulating mind and behavior (Cryan and Dinan, 2012; Bercik and Collins, 2014; Desbonnet et al., 2014; Liu et al., 2015; Evrensel and Ceylan, 2016; Liang et al., 2018b). Among these methods, the first two are only feasible in experimental animals, the third one is usually used in anti-infection, and the last four are all promising in microbiota improvement.

Fecal microbiota transplantation is the process of transplanting feces from a healthy donor to the receiver’s gut in order to recover the impaired intestinal flora. It has been effectively used in the treatment of various diseases including recurrent *Clostridium difficile* infection and inflammatory bowel disease, and its improved model-selective microbiota transplantation- has been put to use (Aroniadis and Brandt, 2013; Zhang et al., 2018). Remolding the gut microbiota through FMT not only recovers digestive function, but also improves the brain and behavior (Evrensel and Ceylan, 2016). Latest research indicates that FMT can be used in the treatment of many brain diseases, such as ASD (Kang et al., 2017), Tourette Syndrome (Zhao et al., 2017), and epilepsy (He et al., 2017).

Probiotics, such as *Lactobacillus* and *Bifidobacterium*, are important components of the gut microbiota, and their related products are widely used in current medications (Kaur et al., 2009; Bested et al., 2013a,b; Sanchez et al., 2017). Dinan et al. (2013) coined the word “psychobiotics” to emphasize on the potential of some probiotics in mental disorder therapy. Animal and clinical studies have identified some psychobiotics that present good antidepressant, anti-anxiety, and/or anti-autism effects. These psychobiotics are likely to work through the regulation of gut microbiota and the improvement of the microbiota–gut–brain axis (Desbonnet et al., 2010; Liang et al., 2015; Mi et al., 2015; Steenbergen et al., 2015; Akkasheh et al., 2016; Pirbaglou et al., 2016; Wallace and Milev, 2017).

Prebiotics mainly include oligosaccharides, unsaturated fatty acids, dietary fibers, and polyphenols (Vyas and Ranganathan, 2012; Liu et al., 2015; Cerdo et al., 2017; Gibson et al., 2017). Studies have found that prebiotics, such as omega-3 fatty acids and oligosaccharides, change the gut microbiota improving the microbiota–gut–brain axis function and symptoms of mental illness subjects (Liu et al., 2015; Burokas et al., 2017; Evans S. et al., 2017; Mika et al., 2017; Robertson et al., 2017). A diet rich in dietary fibers increases gut microbiota diversity, improves the gut barrier, regulates glycometabolism by improving glucose control and insulin sensitivity, modulates lipid metabolism by reducing low-density lipoprotein and cholesterol content, and promotes gut-brain health (Bourassa et al., 2016; Gazzaniga and Kasper, 2016; Koh et al., 2016; Cooper et al., 2017; Gong et al., 2018).

Traditional fermented foods, such as yogurt, natto, and pickles, also regulate the gut microbiota and promote gut-brain health. Diets rich in fermented food, dietary fibers, and unsaturated fatty acids, such as the Mediterranean diet and Japanese diet, also facilitate the proliferation of beneficial microorganisms and improve health and wellbeing (Quirk et al., 2013; Gutierrez-Diaz et al., 2016; Sandhu et al., 2017). The healthy diet probably promotes the function of the microbiota–gut–brain axis and leads to improvements in health and well-being, whereas unhealthy diets including high-fat diets, high-refined carbohydrate diets, and low-MACs diets damage mood and memory (Bereswill et al., 2014; Hu et al., 2014, 2015; Marques et al., 2014; Murphy et al., 2014; Wang et al., 2015; Sandhu et al., 2017). Allen et al. (2017) proposed nutritional psychology to connect the microbiota–gut–brain axis with psychology. In our opinion, nutritional psychology posits that mind and behavior are closely related to the gut microbiota. Food is the most influential factor for the gut microbiota, exerting its influence throughout the whole lifetime. An individual’s diet shapes his or her gut microbiota and regulates gut-brain function. Through the microbiota–gut–brain axis, different types of microbiota exert different influences on the brain and behavior. A healthy diet contributes to a healthy gut microbiota and gut-brain and promotes brain and mental health through the microbiota–gut–brain axis. Meanwhile, an unhealthy diet disturbs the gut microbiota and damages gut-brain function, induces microbiota–gut–brain axis dysfunction and finally harms the brain and well-being.

Dietotherapy is the improvement of health through dietary adjustment. It has been used as an adjuvant treatment for mental disorder therapy for a long time, but it is often doubted for its controversial mechanisms (Lakhan and Vieira, 2008; Lang et al., 2015; Owen and Corfe, 2017). Traditional research has usually focused on the function of certain foods or certain nutrients, such as omega-3 and omega-6 fatty acids (Lakhan and Vieira, 2008; Evans S. et al., 2017; Robertson et al., 2017). Recently, studies have started to pay more attention to the relationship between diet quality and mental illness. Because human beings do not eat just one kind of food, the deficit of one food can have as significant an effect as the excess of another food (Quirk et al., 2013; Lang et al., 2015). Sarris et al. (2015) proposed nutritional psychiatry; they believed that food would play an important part in the prevention and therapy of mental disorders in the future (Sarris et al., 2015; Zepf et al., 2016; Jacka, 2017). With the development of gut-brain psychology and nutritional psychology as the mechanisms of mental disorders and with the role of food in these disorders being clarified, nutritional psychiatry will enter a new stage. Moreover, combining dietotherapy with other interventions, including drug treatment, psychotherapy, and exercise, has shown some good effects in mental therapy (Clarke et al., 2014; Schnorr and Bachner, 2016; Bambling et al., 2017).

## Conclusion and Outlook

Over hundreds of years, psychology has progressed remarkably. Psychologists have intensively studied various mental processes and phenomena, and numerous subdisciplines have sprung up in this process. However, mental disorders remain a problem, and relevant research and therapies lag behind those of other diseases.

This lag reminds us that most psychological research has omitted the fact that the human being is a superorganism. The main part of the superorganism is the microbiota, which accounts for more than 90% of the total genes and total cell numbers in the human body. These microorganisms have coexisted with humans for millions of years and play a vital part in the maturation and function of most human organs. However, since modernization, the commensal microbiota has been continually altered, and in some cases, it has been lost following the dramatic changes in diet, lifestyle, and medication. These alterations have paralleled the transformation of disease patterns in modern society.

The gut microbiota is the most important part of the commensal microbiota, and it can work together with the gut as a whole to respond to endogenous and exogenous signals. The combination of the gut and gut microbiota is called the gut-brain because its activity is partly independent of the brain. The gut microbiota not only regulates the composition and function of the gut-brain, but it also influences the brain and behavior. The abnormal development of the gut microbiota could lead to neurodevelopmental disorders. Various aspects of normal psychology, such as pain perception, emotion, cognition, character, stress management, and social behavior, are impacted by the gut microbiota. Microbiota disturbance can be induced by many factors, including stress, antibiotics, and unhealthy diet, and it could be a direct cause of mental illnesses. Abnormal microbiota is undoubtedly involved in the etiology and pathophysiology of mental disorders, behavioral problems, and neurological diseases, and it will probably be an effective target of future therapy. This viewpoint is strongly supported by the gut microbiota hypothesis, the “old friend” hypothesis, and the leaky gut theory.

The gut-brain is not just a digestive organ but also a neurological, endocrine, and immune organ. The gut microbiota conducts bidirectional information communication with the brain through the microbiota–gut–brain axis; it impacts the host brain and behavior and is also impacted by the host. The microbiota–gut–brain axis mainly consists of the nervous pathway, endocrine pathway, and immune pathway. The nervous pathway mainly operates through neural conductions, neurotransmitters, and through the regulation of neurogenesis, neural apoptosis, and neurodegeneration. The endocrine pathway mainly operates through the neuroendocrine system, neurohormones, and neural active substances. The immune pathway mainly operates through the regulation of innate and adaptive immunity and the peripheral and neural inflammation. Messages transmitted along the microbiota–gut–brain axis are integrated in the brain, the gut-brain, and the three pathways.

The establishment of gut-brain psychology will have a profound influence on psychology and related disciplines. Unlike other psychology subdisciplines, gut-brain psychology will not only promote the progress of fundamental research, but it will also lead to tremendous changes in practical applications. Targeting the microbiota–gut–brain axis to improve brain and behavior will be a research hotspot in neuroscience, psychology, and psychiatry. Improving the gut microbiota through FMT, probiotics, prebiotics, healthy diet, and/or healthy lifestyle to regulate microbiota–gut–brain axis function and promote mental health will be a promising field in the future. Patients suffering from mental disorders or neurological diseases will get help from one or a combination of these interventions. Healthy persons will promote their cognition and resilience from these methods and reduce mental and brain damage by decreasing microbiota disturbances.

## Author Contributions

All the authors listed have contributed to the work. SL conceived the main premises and relationships that are the focus of this review, designed the structure of the paper, wrote the paper, and designed the figures of the paper. FJ conceived the main premises and relationships that are the focus of this review, identified the theoretical relevance of the paper, and edited the final version of the paper. XW offered help in writing the review. All the authors read and approved the final manuscript.

## Conflict of Interest Statement

The authors declare that the research was conducted in the absence of any commercial or financial relationships that could be construed as a potential conflict of interest.
